# Necrotizing enterocolitis leads to disruption of tight junctions and increase in gut permeability in a mouse model

**DOI:** 10.1186/s12887-018-1346-x

**Published:** 2018-11-27

**Authors:** Srikanth Ravisankar, Rodney Tatum, Parvesh M. Garg, Maja Herco, Prem S. Shekhawat, Yan-Hua Chen

**Affiliations:** 1 Department of Pediatrics, Brody School of Medicine, East Carolina University, Greenville, NC 27834 USA; 2 Department of Anatomy and Cell Biology, Brody School of Medicine, East Carolina University, Greenville, NC 27834 USA; 3 Department of Pediatrics, MetroHealth Medical Center, Case Western Reserve University, Cleveland, OH 44109 USA; 4 Present Address: Clinical Neonatologist, Mercy Medical Center, Cedar Rapids, IA 52403 USA; 5 Present Address: Department of Pediatrics, Division Neonatology, University of Mississippi Medical Center, Jackson, Mississippi 39216 USA

**Keywords:** Necrotizing enterocolitis, Tight junctions, Claudin proteins, Biotin tracer molecules, Epithelial barrier function

## Abstract

**Background:**

Necrotizing enterocolitis (NEC) is a leading cause of death in preterm infants. Neonates weighing <1500 grams are at the highest risk for acquiring NEC, with a prevalence of nearly 7-10%, mortality up to 30%, and several long-term complications among survivors. Despite advancements in neonatal medicine, this disease remains a challenge to treat. The aim of this study is to investigate the effect of NEC on gut epithelial tight junctions and its barrier function using a NEC mouse model.

**Methods:**

Three-day old C57BL/6 mouse pups were fed with Esbilac formula every 3 hours and then subjected to hypoxia twice daily followed by cold stress. Dam fed pups from the same litters served as controls. Pups were observed and sacrificed 96 hours after the treatments and intestines were removed for experiments. The successful induction of NEC was confirmed by histopathology. Changes in tight junction proteins in NEC intestines were studied by western blotting and immunofluorescent microscopy using specific protein markers. The gut leakage in NEC was visualized using biotin tracer molecules.

**Results:**

Our study results demonstrate that we induced NEC in >50% of experimental pups, pups lost nearly 40% of weight and their intestines showed gross changes and microscopic changes associated with NEC. There were inflammatory changes with loss of tight junction barrier function and disruption of tight junction claudin proteins in the intestines of NEC mouse model. We have demonstrated for the first time that NEC intestines develop increased leakiness as visualized by biotin tracer leakage.

**Conclusions:**

NEC leads to breakdown of epithelial barrier due to changes in tight junction proteins with increased leakiness which may explain the transmigration of microbes and microbial products from the gut lumen into the blood stream leading to sepsis like signs clinically witnessed.

## Background

Necrotizing enterocolitis (NEC) is a common condition seen in premature neonates born with birth weight <1500g and leads to high morbidity, mortality and long-term complications. The incidence of NEC as reported in the literature varies between 7 to 10% with a high mortality of up to 30% [1]. Survivors of this condition suffer from neurodevelopmental delays [2], poor growth, and cholestasis due short bowel syndrome and may require gut transplantation in rare cases [3].

The cardinal traits considered in the pathogenesis of NEC include prematurity, microbial colonization, genetic predisposition, intestinal immaturity, role of intestinal barriers and response to inflammation by the premature intestine. There is a direct correlation between gestational age and incidence of NEC with highest incidence seen in the most premature neonates [4]. Premature neonates have poorly organized and immature intestinal absorption, digestion and motility patterns [5–9]. Intestinal microbial colonization in these premature neonates is a critical factor and has been demonstrated from early descriptions of this disease from almost four decades ago by Santulli et al [10].

Several animal studies have demonstrated the role of Toll-like receptor 4 (TLR4) in NEC. TLR4 is known to gradually increase in expression in the intestine until term gestation at which point its expression is actively down regulated. It has been reported that TLR4 senses lipopolysaccharides and activation of TLR4 results in local inflammation leading to destruction of the epithelial barrier [11, 12].

Tight junction (TJ) proteins bind the epithelial and endothelial cells together and form a barrier between the intestinal lumen and blood stream [13]. They regulate passage of ions, water and small molecules through the paracellular pathway and thereby maintain distinct body compartments and cellular function [13]. They were first identified in 1986 [14]. Three distinct proteins were identified, Occludin [15], Claudins [16] and junctional adhesion molecules (JAM) [17]. Since then, in addition to discovery of newer TJ proteins, various animal models have helped understand their presence in different organs such as brain, intestines, etc. and demonstrated their critical functions depending on their location in the tissues. Fujita et al. demonstrated the differential expression and localization of various claudins in the intestine of a mouse model [18]. There has been progressively better understanding of the role of TJ proteins in various diseases and the genes [19, 20] encoding for these TJ proteins.

Intestinal permeability is tightly regulated by several TJ proteins, especially the claudins. Claudins are a family of TJ integral membrane proteins with at least 24 members [21]. Claudin proteins are small molecules with ~22 kDa in molecular weight and express different isoforms depending on the tissue and cell type [22]. Claudins show a tissue-specific distribution pattern and are expressed on epithelial linings of the GI tract [18].

It is known that preterm infants have higher intestinal permeability than older children and adults because of various mechanical and nonmechanical factors including TJs that maintain the connections between adjacent cells. The integrity of gut epithelial barrier is absolutely essential for health of GI tract and any disease process which leads to increased permeability to toxins, foreign proteins or translocation of gut bacteria into the circulation starts the process which eventually leads to NEC [23]. Thus, maintenance of gut epithelial barriers by claudins and other barrier function proteins remains central in prevention of NEC. Claudins are known to form pores which are size and charge-selective for various molecules and their normal expression is critical for preservation of epithelial lining. Claudins play a key role in maintaining the epithelial barrier and protecting newborns from development of NEC. The aim of this study is to examine the integrity of the TJs and its barrier function in a NEC mouse model. The gut leakage in NEC intestines was visualized for the first time by biotin tracer molecules, demonstrating the loss of epithelial barrier function in NEC intestines.

## Methods

### Neonatal NEC mouse model

Our animal use protocol was reviewed and approved by the Animal Care and Use Committee of East Carolina University (AUP#A182). All animals were housed in the facility maintained by East Carolina University, which provides in-house veterinary care. The animals were kept in rooms at 22°C with a 12-h light-dark cycle. We performed the animal experiments according to the guidelines of East Carolina University and the National Institute of Health on animal care and use. These guidelines are very similar to ARRIVE guidelines practiced by investigators in the European Union [24]. Every effort was made to minimize the pain and discomfort to the animals during the experiments.

The 3-day old C57BL/6 pups were divided into 2 groups: dam fed control group (n=13) and experimental group (n=13). The latter group was fed with 30-50 μl 33% Esbilac formula (Pet-Ag, New Hampshire, IL) every 3 hours for 96 hours using a 1.9 Fr feeding tube, subjected to hypoxia (100% N_2_ for 60 seconds) and cold stress (4°C for 10 min) twice a day for four days. Pups were nursed in an incubator (37°C) during the 4-day period and provided high calorie formula. Both the experimental and dam fed control pups were euthanized at 96 hours after start of the experiment. The gastrointestinal tract was carefully dissected and visually evaluated for signs of NEC (areas of bowel necrosis, intestinal hemorrhage). NEC was confirmed by histopathology using published tissue injury guidelines in mice [25, 26]. Intestinal samples were collected for histopathological and immunofluorescent studies.

### Histology examinations

Intestinal tissues isolated from control and NEC pups were fixed in 10% formalin solution. After washed with phosphate buffered saline (PBS), the fixed tissues were processed through graded ethanol solutions. The 5 μm tissue sections were cut using a cryostat. Hematoxylin & Eosin (H&E) staining was performed according to the published methods [27].

### Western blot analysis

Intestinal tissues from control and NEC pups were minced on ice, homogenized in RIPA buffer (1% Triton X-100, 0.5% sodium deoxycholate, 0.2% SDS, 150 mM NaCl, 10 mM Hepes, pH7.3, 2 mM EDTA, 10 μg/ml each of chymostatin, leupeptin and pepstatin A) by passing 10 times through a 22-gauge needle, and centrifuged at 15,000 g for 30 minutes at 4°C to obtain tissue lysates. The total protein concentration of each sample was measured using the BCA protein assay kit (Pierce, Rockford, IL, USA) and adjusted to equal concentration (2 mg/ml). Proteins in the SDS sample buffer were separated by SDS-PAGE and transferred to nitrocellulose membranes. After blocking by 5% nonfat dry milk, the membranes were incubated with primary and secondary antibodies, respectively. The signals were detected by enhanced chemiluminescence (Amersham, Arlington Heights, IL, USA).

### Immunofluorescence microscopy

Intestinal tissues isolated from control and NEC pups were frozen in liquid nitrogen. The 5 μm frozen sections were cut using a cryostat. The frozen sections were fixed in 100% acetone and blocked with 5% BSA. The primary and secondary antibodies were applied to the intestinal tissues, respectively. The immunofluorescent signals were analyzed using a Zeiss Axio Imager M2 microscope (Carl Zeiss, Thornwood, NY).

### Tight junction permeability assay *in vivo*

This TJ *in vivo* permeability assay using biotin has been published previously [28]. Biotins are small water-soluble molecules. They are membrane impermeable reagents and allow efficient labeling of proteins and primary amine - containing macromolecules on the cell surface. Biotin reagents will not diffuse through the intercellular space if TJ is intact. However, if TJ structure is disrupted, the biotin molecule will penetrate into the intercellular space.

To examine the TJ barrier function, the pups were anesthetized by intraperitoneal injection with 0.05ml/10g body weight of Ketamine (18mg/ml) and Xylazine (2mg/ml) at the end of NEC experiments. After ensuring the pup was in deep anesthesia with paw pinch, the abdominal cavity was opened by a surgical scissor. The Sulfo-NHS-Biotin (MW: 556.59) diluted in 1 mg/mL PBS was injected into the lumen of control and NEC small intestines using a low-pressure syringe pump (Harvard Apparatus). The injection was conducted at a rate of 50 μl/min through a 25G needle connected to a polyethylene tubing. Ten minutes after biotin injection, intestines were dissected and embedded in O.C.T. compound, then processed for immunofluorescence light microscopy [28]. Biotin was detected by Texas red-conjugated streptavidin.

After the tissue collection, pups were euthanized by decapitation while under anesthesia. All these procedures were approved by the East Carolina University (ECU) Animal Care and Use Committee and conducted in compliance with guidelines from the National Institute of Health and ECU on laboratory animal care and use.

### Statistical analysis

Statistical analysis was performed using either Origin8 (OriginLab, MA) or SIGMAPLOT (Systat Software, Inc. CA) softwares. The differences between two groups were analyzed using the unpaired Student’s *t*-test. The *p*-value of < 0.05 will be considered significant.

## Results

### Gross and histological examinations of intestines

**Fig. 1:**
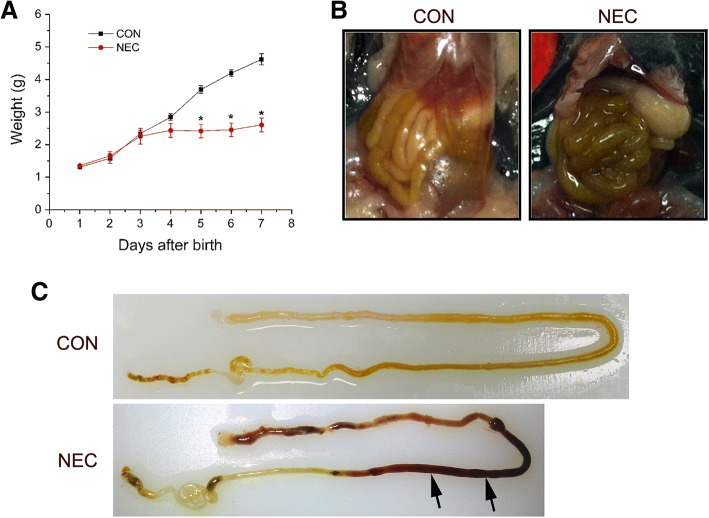
Establishment of necrotizing enterocolitis (NEC) mouse model. **a**: Weight measurement of dam-fed (CON) or NEC-treated (NEC) pups. The treatment started at day 3 and was continued for 4 days. The data represent means ± s.e.m. from three independent experiments. Student’s *t*-test was used for statistical analysis. A *P* value of < 0.05 was considered significant (*). N=10 pups for each group. **b**: Comparison of control and NEC intestines *in situ* at the end of the experiments. The NEC intestine showed the swollen and discolored appearance. **c**: The control and NEC intestines after removal from the body. The NEC intestine displayed the severe hemorrhage as indicated by arrows

**Fig. 2:**
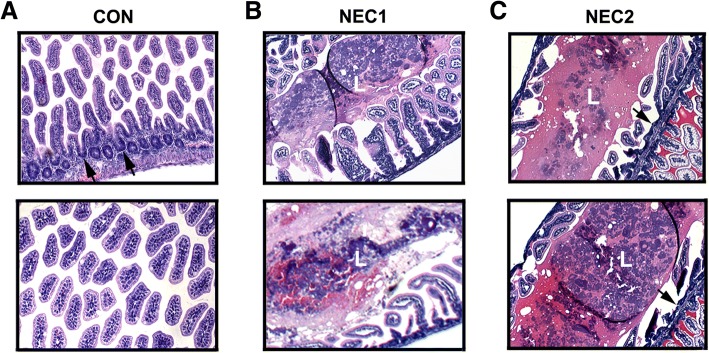
H& E staining of control and NEC intestines. **a**: Tissue sections from representative control intestines shows the normal villous structure with intact crypt region (arrows). **b** and **c**: Tissue sections from representative NEC intestinal samples. The induction of NEC led to stunting of villi and disrupted villous structure with sloughing of epithelial cells into the lumen (L). The hemorrhage is evident in both B and C NEC intestinal samples. Original magnification: 200×

After 4 days of treatments, about 50% of thirteen-treated pups developed NEC compared to none in the dam fed group. The NEC pups were smaller in size and had an average of 42% reduction of the weight compared to the control pups at the end of the treatment (Fig. 1a). The NEC intestines showed the clinical sign of the disease and were inflamed and swollen in appearance (Fig. 1b). Grossly necrotic bowel was seen mainly in the small intestines, and part of the large intestine was also involved (Fig. 1c). On microscopic examination, NEC intestines showed the clear evidence of bloody necrotic changes as indicated by the arrows when compared to the controls (Fig. 1c). The NEC phenotype was confirmed by histopathology examinations using a standard histopathological grading established to diagnose NEC [26]. Control intestines showed the intact villus structure and submucosa (Fig. 2a) while NEC intestines had a disrupted villous mucosal structure. Some NEC villi displayed complete sloughing of epithelial cells with breach of epithelium barrier (Fig. 2b and c).

### Altered expression and localization of TJ proteins and elevation of inflammation marker proteins in NEC intestines

**Fig. 3:**
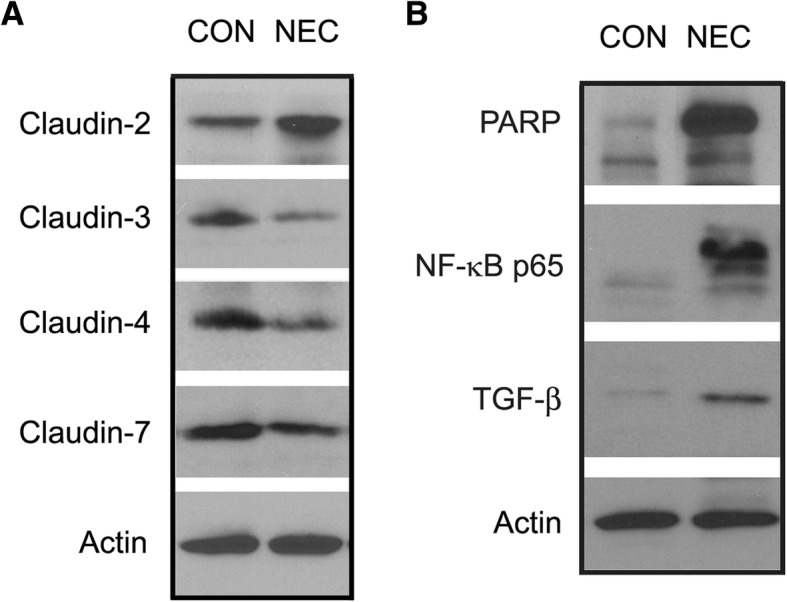
Protein expression levels of control and NEC intestines. **a**: Representative western blot membrane showing claudin-2, -3, -4 and -7 expression levels in control (CON) and NEC intestines. Intestinal tissues from 7-day control and NEC pups were collected at the end of experiments. Tissue lysates were solubilized in RIPA buffer and subjected to western blotting. **b**: PARP, NF-κB and TGF-β expression levels in control (CON) and NEC intestines. A total of 30 μg protein for each sample were loaded on the SDS-polyacrylamide gel. Membranes were blotted against specific antibodies. Actin served as a loading control. Three independent experiments were performed

Western blot analysis showed an altered expression of TJ claudin proteins. There was an increased expression level of claudin-2 and decreased expression levels of claudin-3, -4, and -7 in NEC intestines compared to those of controls (Fig. 3a). In addition, the expression levels of PARP, a protein that can induce a programmed cell death, as well as inflammatory marker proteins NF-kB and TGF-β were all up-regulated in the NEC intestines compared to the control intestines (Fig. 3b).

**Fig. 4:**
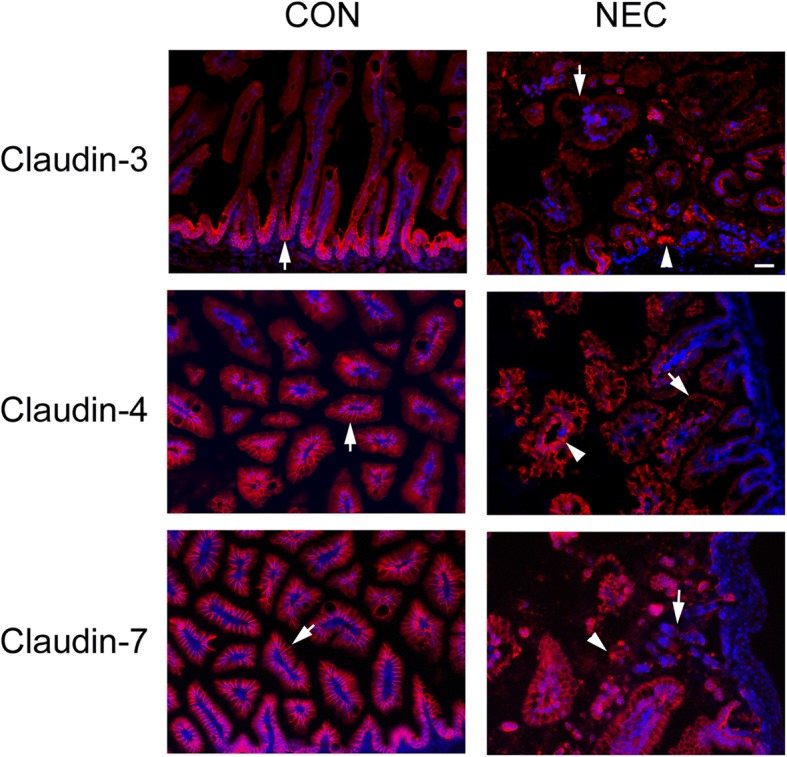
Immuno-localization of claudin proteins in control and NEC intestines. Representative images of intestines from 7-day control and NEC pups were dissected from the body and frozen in liquid nitrogen. Frozen sections were immunostained with anti-claudin-3, or -4, or -7 antibodies and detected by Cy3-conjugated secondary antibody. Nuclei were stained with DAPI (blue). The claudin signals were localized at cell-cell contact area as indicated by arrows in controls (CON). Arrows were pointed to the reduced signals in NEC samples and arrowheads in NEC showed the aggregated staining pattern. Scale bar: 50 μm

Immunofluorescent microscopy results indicated reduced immunostaining signals for claudin-3, -4, and -7 as indicated by arrows in NEC intestines (Fig. 4, arrows, NEC) compared to those of control intestines (Fig. 4, arrows, CON). In addition, the aggregated signals of claudin-3, -4, and -7 could be clearly observed in NEC intestinal tissues (Fig. 4, arrowheads in NEC).

### Disruption of TJ barrier function in NEC intestines

**Fig. 5:**
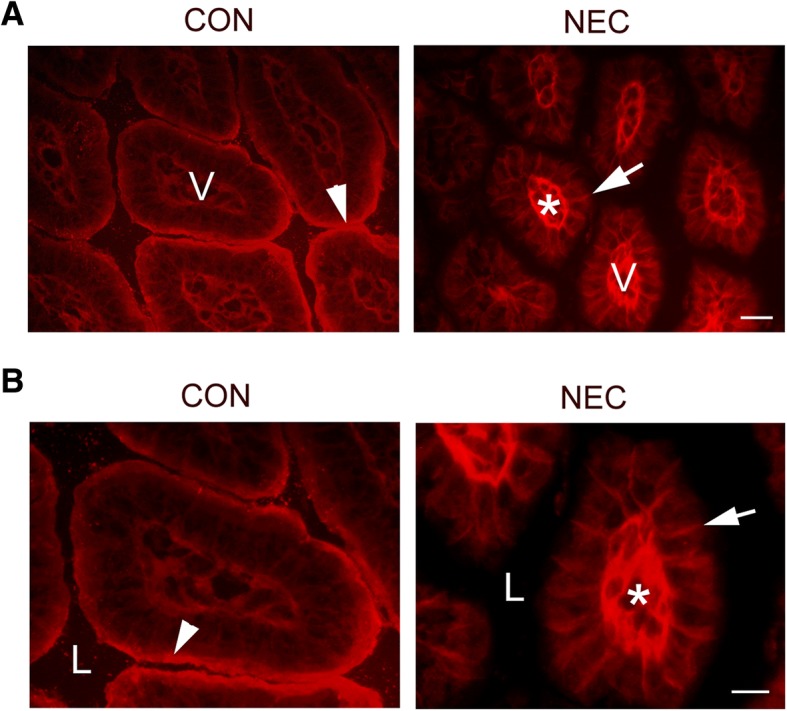
Biotin permeability assays in control and NEC intestines. **a**: Sulfo-NHS-LC-Biotin was injected into the intestinal lumen of 7-day old control (CON) or NEC pups. The tissue sections were stained with Texas red-conjugated streptavidin. Biotin was mainly kept at the epithelial surface of control intestines (arrowhead) due to the intact epithelial barriers. No barrier leakage was observed in control intestines. In contrast, the leakage was clearly detected in NEC intestines. Biotin stained the intercellular space (arrow) and connective tissue (Asterisk). V: Villus. **b**: The higher magnification of the images. L: Lumen. Scale bar: 50 μm in A and 20 μm in B

To study whether NEC intestines have a leaky TJ or not, biotin *in vivo* detection assay was employed. The biotin staining signal was examined under the immunofluorescent light microscope. We found that NEC intestines displayed a diffusion of biotin signal through the intercellular space (Fig. 5, arrows in NEC). On the other hand, the control intestines showed no sign of leakage of TJ barrier. The biotin molecules were kept at the intestinal surface facing the lumen where biotin was applied (Fig. 5, arrowhead, CON). The strong biotin staining signal marked by asterisk reflected the accumulation of biotin molecules into the intestinal tissue (Fig. 5, asterisk in NEC).

## Discussion

The conundrum of NEC will continue to be an important challenge both for the practicing clinician and the basic science scientists. Though numerous studies have contributed to various aspects of pathogenesis of NEC, the fundamental etiopathogenesis remains a big unanswered question. This study focused on the role of TJ integral membrane protein claudins which play an essential role in cellular integrity and barrier function in the intestine.

The integrity of TJs is vital in maintaining intestinal epithelial homeostasis both at physiological condition and in disease state. Our NEC mouse model indicates an altered expression in the claudins where there is an increase in expression of claudin-2 and with a decreased expression of claudin-3, -4 and -7. It is well known that claudin-2 creates cation selective pores and increases cation permeability in epithelia of various tissues including intestines [29–31]. We observed an increased expression of claudin-2 in our NEC intestines which is consistent with the hypothesis of leaky TJs in NEC. The function of claudin-3 and -4 in the intestine is well established as a barrier and its down-regulation observed in our NEC mouse model explains the loss of barrier function [32]. It has been reported that claudin-7 is involved in cell-matrix interactions [33, 34]. Reduced expression of claudin-7 could contribute to the loss of tissue integrity. Claudins have been studied in human subjects and increased gut permeability has been demonstrated in cases with NEC [35, 36]. Thus, our study provides the molecular basis behind the observed clinical effect. Further research could focus on modulating TJ protein expression in the preterm neonatal gut to prevent NEC.

Biotins are membrane impermeable reagents and therefore, will not diffuse through the intercellular space if TJ is intact. However, if TJ structure/function is disrupted, the biotin molecule will penetrate into the intercellular space. Biotin permeability assay has been successfully applied to many different *in vivo* systems to examine the TJ permeability barrier, such as the blood brain barrier in claudin-5-deficient mice [37], the blood brain barrier in adult zebrafish [38], and the pathogen-infected intestinal epithelia [39]. Our study also demonstrates the increased gut permeability in NEC intestines. Disruption of TJ barrier function in NEC intestines is visualized for the first time by biotin *in vivo* detection assay. Disruption of epithelial barrier has been studied in human infants using indirect markers of epithelial cells like fatty acid binding protein (I-FABP). We did not use this marker in our mouse model since intestinal injury was grossly visible in affected animals.

Leaky TJs in NEC induce inflammation in the gut as reported by other studies [11, 40–43]. Our current study also observed increased signals for inflammatory markers, NF-kB and TGF-β. Both NF-kB and TGF-β are involved in the regulation of inflammatory processes. In addition, it is worth pointing out that NEC induction not only induces inflammatory response in GI tract, but also in kidneys as we have previously reported [44]. Further studies are necessary to better understand the relationship of a disrupted TJ structure/function with inflammation and severity of the NEC and whether altered expression of TJ protein claudins can be used as a potential biomarker for NEC.

The major limitation of our current study is that it is an animal study conducted using mice subjected to feeding a hyperosmolar formula and periods of hypoxia which is not exactly the same pathology seen in human neonates where infection starts the NEC process in most cases. But, at the same time inflammation which is the end result, was witnessed in our mouse model as well. Thus, there are similarities between clinical NEC and what we witnessed in our mouse model. Our report suggests that by modulating the expression of certain TJ proteins and control of the inflammatory process could potentially change the course of NEC. However, this goal will require further basic and clinical research.

## Conclusions

We have successfully induced NEC in a mouse model where there was widespread inflammation and histological changes like those seen in human neonates. Inflammation was associated with increased expression of NF-κB, TGF-β and PARP. There was increased expression of Claudin-2 along with down-regulation of Claudins-3, -4, and -7 which explains the reason for breakdown of the epithelial barrier. We have demonstrated increased leakiness through the epithelial lining using biotin tracer, that explains the clinical sepsis like features witnessed in neonates. Thus, our report provides the molecular basis behind the clinical picture of NEC and identifies molecules where future therapies could be targeted.

## Abbreviations

JAM: Junction adhesion molecules
NEC: Necrotizing enterocolitis
NF-κB: Nuclear factor κB
PARP: Poly (ADP-ribose) polymerase
TGF-β: Transforming growth factor-β
TJ: Tight junction
TLR: Toll-like receptor
